# Immunophenotypical Switch versus Tumor Heterogeneity in a Patient with HIV-Associated Diffuse Large B-Cell Lymphoma

**DOI:** 10.4061/2011/563216

**Published:** 2010-10-05

**Authors:** Jorge J. Castillo, Tina Rizack, Diana Treaba

**Affiliations:** ^1^Division of Hematology and Oncology, The Miriam Hospital, The Warren Alpert Medical School of Brown University, 164 Summit Avenue, Providence, RI 02906, USA; ^2^Department of Pathology, Rhode Island Hospital, The Warren Alpert Medical School of Brown University, Providence, RI 02903, USA

## Abstract

Patients with HIV/AIDS have a higher risk of developing aggressive B-cell lymphomas, such as diffuse large B-cell lymphoma (DLBCL). Lymphomas are rather heterogeneous in nature and in a few cases can switch their genetic or immunohistochemical phenotype, transform into other lymphomas or carry more than one malignant clone. In this report, we present the case of a 47-year-old man with HIV infection who was diagnosed with an apparent low-risk, early-stage DLBCL, but became refractory to therapy while undergoing treatment with rituximab-containing chemotherapy. We postulate that the development of his refractory disease occurred in the context of an immunohistochemical switch or the surge of a clone refractory to therapy. This phenomenon was not associated with a superinfection with EBV or HHV-8.

A 47-year-old HIV-positive male presented complaining of one-week course of right arm pain and swelling within the right axillary area. He denied fevers, night sweats, weight loss, or trauma to his arm. His past medical history was significant for HIV diagnosed 10 years earlier with a recent CD4 count of 456 cells/mm^3^. He worked as a cook, lived with his male partner, was on no antiretroviral therapy (ART), drank rarely, and did not engage in the use of illicit drugs. Physical exam revealed a 4.5-cm right axillary mass and a palpable mass within the right pectoralis region that was tender on palpation. There was no hepatosplenomegaly and no other abnormalities on physical examination. Laboratories revealed a white blood cell count (WBC) of 5.7 × 10^9^/L with a normal differential, hemoglobin of 14.3 g/dL, platelet count of 166 × 10^9^/L, and LDH of 265 IU/L. A computerized tomography (CT) scan of the chest showed a markedly enlarged right pectoralis minor muscle with an adjacent enlarged right axillary lymph node measuring 5.2 × 3.0 cm. CT scans of the abdomen and pelvis were unremarkable. 

An excisional biopsy of a right axillary lymph node was performed. On gross pathological examination, large atypical cells were seen with features of centroblasts and immunoblasts along with numerous mitotic figures. Immunohistochemically, the large cells are positive for CD20, PAX5, and bcl-2, weakly positive for bcl-6, CD30, and negative for CD3, CD5, CD10 and MUM-1 (Figure 1). A bone marrow biopsy and aspirate showed normocellularity and trilineage hematopoiesis with no evidence of lymphoma. Patient was diagnosed with stage IIA diffuse large B-cell lymphoma of a germinal center (GC) subtype. His International Prognostic Index (IPI) score was 1. He was initiated on chemotherapy with R-CHOP (rituximab, cyclophosphamide, doxorubicin, vincristine, and oral prednisone) every 3 weeks. His LDH was normalized after the first cycle of chemotherapy and after the second cycle a CT scan of the chest showed significant decrease in size of the prior lymphadenopathy. 

After the third cycle of chemotherapy, he developed a vesicular rash in the right pectoral area felt to be herpes zoster and treated with valacyclovir. Despite antivirals, the rash progressed. ART was then initiated with efavirenz, emtricitabine, and tenofovir when his CD4 count was 309 cells/mm^3^. The rash progressed more rapidly and developed a more nodular appearance along with the simultaneous development of progressive right axillary lymphadenopathy, and right upper extremity, chest wall, and scrotal edema. A PET/CT scan showed interval decrease in the size of initial bulky chest and axillary adenopathy but revealed extensive right supraclavicular, axillary, and right lateral wall FDG-avid disease as well as FDG-avid cutaneous and subcutaneous nodular thickening of the right anterolateral chest wall. The patient was referred to a surgeon for a skin and lymph node biopsy. 

While awaiting results of the biopsies, he developed left-sided facial numbness and presented to the emergency room where a noncontrast cat scan of his head was negative, and a lumbar puncture (LP) was performed. Results of his LP showed a protein of 87 mg/dl (normal range 15–45 mg/dl) and glucose of 58 mg/dl (normal range 38–85 mg/dl) as well as rare atypical cells felt to be consistent with lymphoma. An MRI of the brain with gadolinium demonstrated abnormal soft tissue enhancement of the left Meckel's cave and right internal auditory canal, highly suspicious for lymphomatous meningitis. Intrathecal methotrexate was initiated. His left facial weakness progressed and the patient developed ptosis and blurry vision of the left eye, which was resolved after the second injection of intrathecal methotrexate. 

Pathological examination of his biopsy showed cells with large nuclei and open chromatin, one to multiple conspicuous nucleoli, and scant cytoplasm. There were also numerous apoptotic cells. By immunohistochemistry, this neoplastic lymphoid population was negative for CD20, bcl-6, and CD10 but uniformly positive for CD45, CD43, CD30, and MUM-1 (Figure 2), and had a variable PAX-5 and bcl-2 positivity. These neoplastic B cells had a high proliferation rate (MIB-1 90%–100%) but were negative for CD3, CD4, CD8, cyclin-D1, CD15, and anaplastic lymphoma kinase (ALK-1). An anti-EBV latent membrane protein antibody was negative. Molecular studies were also performed and detected the presence of IgH gene rearrangement, however, there were no T-cell receptor gene rearrangements, no bcl-2 translocation, and no EBV DNA sequences detected. FISH studies were negative for the presence of MYC rearrangements. He was diagnosed with relapsed stage IV DLBCL of a nongerminal center (NGC) immunophenotype. An Ommaya reservoir was placed for biweekly methotrexate and a port-a-cath placed for rituximab with infusional EPOCH (etoposide, vincristine, doxorubicin, cyclophosphamide, and prednisone). As his neurological symptoms, rash, and lymphadenopathy had improved, the patient was discharged home and returned to clinic for biweekly intrathecal methotrexate. Three weeks later, the patient was admitted for his second cycle, during which he developed worsening pain and swelling on his right side. The patient and his partner decided to pursue palliative radiation to his right chest wall. Shortly thereafter, the patient developed a sharp decline in his mental status. A head CT scan showed a suprasellar mass and CNS extension of his DLBCL at which point the patient and his partner elected to pursue hospice care.

DLBCL is the most common lymphoma variant seen in the general population, accounting for approximately 25%–30% of all cases [1]. Individuals infected with HIV are at a higher risk of developing DLBCL than the general population. With the advent of HAART, the absolute incidence of DLBCL in HIV-positive individuals seems to have decreased [2]; however, the relative incidence has probably increased due to deeper impact on the incidence of other common malignancies such as Kaposi sarcoma [3]. The treatment of DLBCL in HIV-infected individuals consists on combination chemotherapy; RCHOP is the standard of care for immunocompetent patients with DLBCL [4, 5]. A phase III trial showed no survival benefit in HIV-positive DLBCL patients with the addition of rituximab to CHOP [6]; however, with better prophylactic schemes, HIV-positive patients with a CD4 count >100 cells/mm^3^ can derive benefit from the addition of rituximab [7]. 

There are few studies showing phenotypical and histological changes in patients with lymphomas after exposure to rituximab [8]. Rituximab is a chimeric anti-CD20 monoclonal antibody; hence, it is not surprising for it to downregulate CD20 expression within the tumoral cells. This effect is reflected by the CD20negativity seen in the second sample from our patient. This phenomenon is commonly seen on biopsies of relapsed or refractory cases of lymphoma [9]; however, the loss of CD20 has not been formally associated with a worse outcome [10]. The IPI score at diagnosis in our patient was 1 (elevated LDH), implying a low-risk disease with an estimated 5-year overall survival and complete response rate of 73% and 87%, respectively. Our patient failed to obtain a complete response to RCHOP and progressed while on active treatment. A rebiopsy showed a similar histology but with a different phenotype that was not limited to the loss of CD20. Based on the Hans classification [11] our patient presented initially with a CD20-positive DLBCL of a GC origin (CD10 negative, bcl-6 positive, MUM-1 negative), but on biopsy of his refractory disease his phenotypic profile switched to a CD20-negative NGC (CD10 negative, bcl-6 negative, and MUM-1 positive) DLBCL. A literature search failed to find similar cases in the literature. Hence, we are unsure of the clinical significance of this immunophenotypical switch but believe it could correlate with this patient's aggressive clinical course, despite his apparent initial low-risk disease.

There is, however, another potential explanation for our findings. Our patient could have had 2 different clones, the GC one that responded initially to RCHOP and the NGC one that was refractory to RCHOP and finally progressed while on treatment. The fact that both biopsies were taken from the same anatomical area makes this possibility, although not impossible, less likely. Molecular studies evaluating IgH gene rearrangements could have been helpful to prove a clonal relationship between the two processes; unfortunately, the IgH PCR study at diagnosis was negative while the second IgH PCR showed one band consistent with the expansion of a single lymphoid clone. 

As a side point, our patient did not receive HAART at initial DLBCL diagnosis because his CD4+ cell count was 456 cells/mm^3^. Later on, he was started on HAART after his refractory disease was identified when his CD4+ cell count was 309 cells/mm^3^. It is unclear if the lack of use of HAART could have played a role in the refractoriness of his disease. However, few studies have shown that stopping HAART temporarily while undergoing chemotherapy is safe and does not seem to affect survival in patients with HIV-associated DLBCL [12].

Our case highlights the biological plasticity inherent to lymphoproliferative disorders, in which spontaneous (i.e., Richter's transformation) and/or therapy-induced (i.e., post rituximab) changes are not uncommon. We present a patient with HIV-positive DLBCL that became refractory to RCHOP despite his apparent low-risk disease, maybe associated to an immunophenotypical switch from an GC to a NGC profile or the development of a chemotherapy-refractory clone.

## Figures and Tables

**Figure 1:**
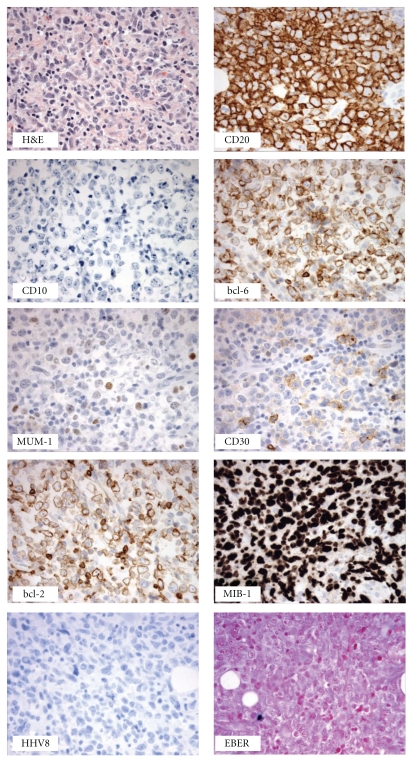
CD20-positive diffuse large B-cell lymphoma with a germinal center immunophenotypical profile (initial excisional biopsy).

**Figure 2:**
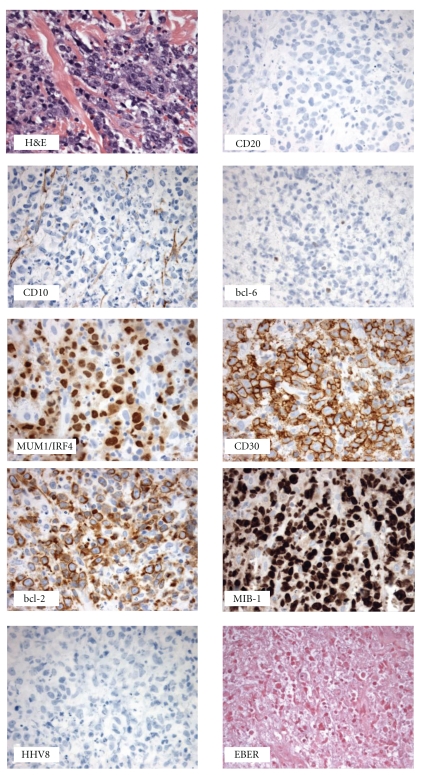
CD20-negative diffuse large B-cell lymphoma with a nongerminal center immunophenotypical profile (second excisional biopsy).
